# The time-varying prognostic value of stenosis and plaque burden in coronary artery disease

**DOI:** 10.1093/ehjci/jeag022

**Published:** 2026-01-28

**Authors:** Ruurt A Jukema, Teemu Maaniitty, Nick S Nurmohamed, Pieter G Raijmakers, Roel Hoek, Roel S Driessen, R Nils Planken, Jos Twisk, Pim van der Harst, Maarten J Cramer, Antti Saraste, Paul Knaapen, Juhani Knuuti, Ibrahim Danad

**Affiliations:** Department of Cardiology, Amsterdam Cardiovascular Sciences, Amsterdam UMC, Vrije Universiteit Amsterdam, De Boelelaan 1117, Amsterdam 1081 HV, The Netherlands; Turku PET Centre, University of Turku, Turku, Finland; Clinical Physiology, Nuclear Medicine and PET, Turku University Hospital, Turku, Finland; Department of Cardiology, Amsterdam Cardiovascular Sciences, Amsterdam UMC, Vrije Universiteit Amsterdam, De Boelelaan 1117, Amsterdam 1081 HV, The Netherlands; Department of Vascular Medicine, Amsterdam UMC, Vrije Universiteit Amsterdam, Amsterdam, The Netherlands; Division of Cardiology, The George Washington University School of Medicine, Washington, DC, USA; Department of Radiology, Nuclear Medicine and PET Research, Amsterdam UMC, Vrije Universiteit Amsterdam, Amsterdam, The Netherlands; Department of Cardiology, Amsterdam Cardiovascular Sciences, Amsterdam UMC, Vrije Universiteit Amsterdam, De Boelelaan 1117, Amsterdam 1081 HV, The Netherlands; Department of Cardiology, Amsterdam Cardiovascular Sciences, Amsterdam UMC, Vrije Universiteit Amsterdam, De Boelelaan 1117, Amsterdam 1081 HV, The Netherlands; Department of Radiology and Nuclear Medicine Amsterdam Cardiovascular Sciences, Amsterdam UMC Location UvA, Amsterdam, The Netherlands; Department of Epidemiology and Data Science, Amsterdam UMC, Vrije Universiteit Amsterdam, Amsterdam, The Netherlands; Department of Cardiology, University Medical Centre Groningen, University of Groningen, Groningen, The Netherlands; Department of Cardiology, Division of Heart and Lungs, Utrecht University, Utrecht University Medical Center, Heidelberglaan 100, 3584 CX, Utrecht, The Netherlands; Turku PET Centre, University of Turku, Turku, Finland; Heart Center, Turku University Hospital, Turku, Finland; Department of Cardiology, Amsterdam Cardiovascular Sciences, Amsterdam UMC, Vrije Universiteit Amsterdam, De Boelelaan 1117, Amsterdam 1081 HV, The Netherlands; Turku PET Centre, University of Turku, Turku, Finland; Clinical Physiology, Nuclear Medicine and PET, Turku University Hospital, Turku, Finland; Department of Cardiology, Amsterdam Cardiovascular Sciences, Amsterdam UMC, Vrije Universiteit Amsterdam, De Boelelaan 1117, Amsterdam 1081 HV, The Netherlands; Department of Cardiology, Division of Heart and Lungs, Utrecht University, Utrecht University Medical Center, Heidelberglaan 100, 3584 CX, Utrecht, The Netherlands

**Keywords:** CCTA, plaque burden, diameter stenosis, prognostic value

## Abstract

**Aims:**

Conflicting results have been reported on the prognostic value of coronary stenosis grade and plaque burden. We aimed to investigate the time-varying risk for cardiovascular events associated with diameter stenosis (DS%) and plaque burden.

**Methods and results:**

Patients without a documented cardiac history who underwent coronary computed tomography angiography for suspected coronary artery disease were included. The most severe DS% and plaque burden, defined as percentage atheroma volume (PAV), were used for analysis. The primary endpoint was a composite of all-cause mortality and non-fatal myocardial infarction. For analysis, the maximal follow-up time was 8 years. Among 2819 patients [mean age 62 ± 10; 1245 (45%) male], 235 events occurred during a median follow-up of 6.9 years. Cox models including cardiovascular risk factors, DS%, and PAV demonstrated that DS% but not PAV was predictive for short-term events at 1-year follow-up [adjusted hazard ratio (aHR) 1.028, 95% confidence interval (CI) 1.013–1.044 vs. 1.015, 95% CI 0.978–1.053]. In contrast, PAV but not DS% was predictive for long-term events at 8-year follow-up (aHR 1.035, 95% CI 1.021–1.050 vs. 1.005, 95% CI 0.999–1.012). The predictive value of DS% was stronger before than after 1 year of follow-up (aHR <1 year 1.027, 95% CI 1.012–1.042 vs. aHR 1–8 years 1.001, 95% CI 0.994–1.008; *P* < 0.01 for difference), while the predictive value of PAV did not significantly change (*P* = 0.12).

**Conclusion:**

Coronary diameter stenosis holds the highest prognostic significance for short-term cardiovascular events, while plaque burden predicts events in the long term.

## Graphical Abstract

**Graphical Abstract:**
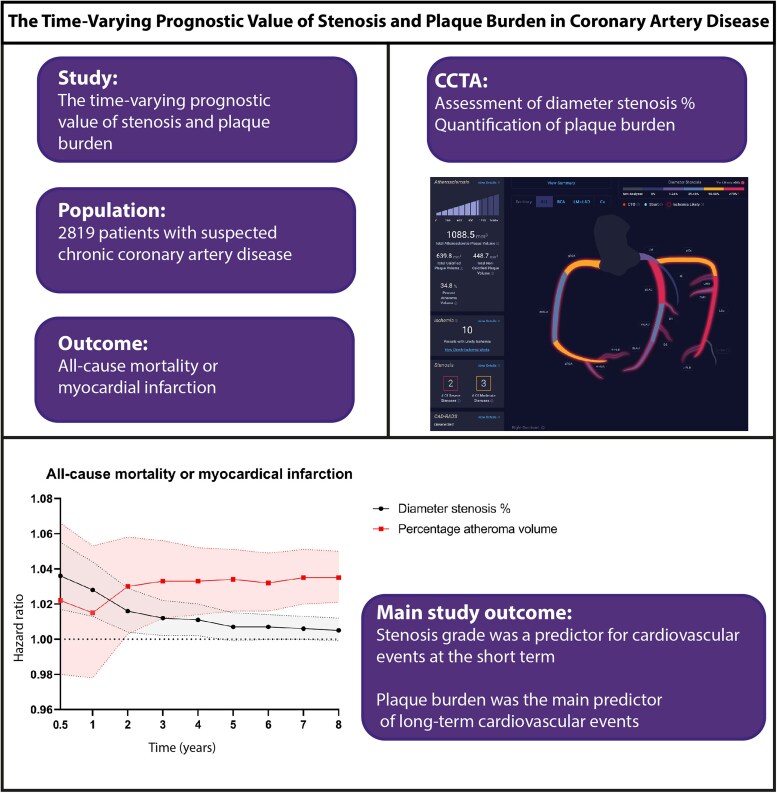

**See the editorial comment for this article ‘Coronary atherosclerosis: plaque volume determines long-term prognosis’, by A. Schmermund**  ***et al*****., https://doi.org/10.1093/ehjci/jeag040.**

## Introduction

Coronary computed tomography angiography (CCTA) has emerged as a pivotal tool in the non-invasive assessment of coronary artery disease (CAD), providing detailed anatomical information regarding the presence and extent of coronary atherosclerosis.^1^ CCTA is renowned for its excellent prognostic value being able to differentiate patients with or without atherosclerosis.^2^ Historically, the focus of CCTA was to detect high-grade stenosis, even though the occurrence of acute coronary syndromes predominantly originates from non-obstructive plaque on baseline CCTA.^3^ This observation sparked interest in quantifying total plaque burden in addition to stenosis grade, which is since recently facilitated by machine-learning quantitative plaque tools.^4,5^

Conflicting results have been reported regarding the predictive value of stenosis grade and plaque burden. In both a large observational CCTA study and a sub-study of the SCOT-HEART trial, stenosis grade did not confer incremental prognostic value after correction for plaque burden.^6,7^ In contrast, stenosis grade contributed significantly to improved risk prediction in addition to plaque burden in a sub-study of the CONFIRM trial.^8^ Interestingly, apart from the disparate plaque burden assessment methodologies, an important difference between those studies is the follow-up time (5 vs. 2 years). Accordingly, the findings should be considered within the framework of dynamic risk stratification, acknowledging that both the prognostic value of patient-specific risk factors and imaging markers, such as stenosis severity and plaque burden, may evolve over time. As such, this study aimed to investigate the time-varying prognostic value of coronary stenosis and plaque burden for the prediction of cardiovascular events in symptomatic patients evaluated for suspected CAD.

## Methods

### Patient population

A total of 3201 symptomatic patients from Turku University Hospital and Amsterdam University Medical Centers, location VU University Medical Center, who underwent CCTA because of suspected stable CAD between 2007 and 2016, were assessed for inclusion. Patients with prior documented CAD were excluded. The treatment strategy following CCTA was left to the discretion of the referring physician, who was aware of the CCTA findings. Early revascularization was defined as having occurred in the first 6 months following CCTA. The need for written informed consent was waived by the ethics committees of the Hospital District of Southwest Finland and Amsterdam University Medical Centers due to the retrospective nature of the study. The study adhered to the principles of the Declaration of Helsinki.

### Coronary computed tomography angiography

Patients with a regular heart rate (target rate below 65 bpm, either spontaneous or after administration of oral and/or intravenous metoprolol) underwent CCTA using a ≥ 64 slice scanner. Standard scanning protocols were applied, with a tube voltage of 100–120 kV and a tube current of 600–1000 mA. Detailed scanning protocols were described in detail before.^9,10^

### Atherosclerosis imaging-quantitative coronary computed tomography

A US Food and Drug Administration-approved artificial intelligence based software, atherosclerosis imaging-quantitative coronary computed tomography (AI-QCT, Cleerly Inc., Denver, CO, USA), was used to analyse the CCTA images.^5^ The AI-QCT software utilizes validated convolutional neural networks for image quality assessment, coronary segmentation, vessel contour determination, lumen wall evaluation, and plaque quantification and characterization. First, the algorithm creates a centreline, lumen, and outer vessel wall contours for every phase available. Subsequently, the algorithm uses the 2 optimal series for analysis for each coronary artery. Following automated segmentation and labelling of all coronary arteries, plaques are characterized and quantified based on Hounsfield unit (HU) attenuation. Finally, the analysis was supervised by a radiologic technologist for quality assurance review. AI-QCT analyses were performed on a per segment basis using the modified 18-segment Society of Cardiovascular Computed Tomography model.^11^ Segments were evaluated for the presence of coronary atherosclerosis, defined as any tissue structure >1 mm^2^ within the coronary artery wall that was differentiated from the surrounding epicardial tissue, epicardial fat, or the vessel lumen itself. Only coronary segments with a diameter ≥1.5 mm were included for analysis. Coronary diameter stenosis (DS) percentage was adjudicated on a per-vessel basis. The maximum DS percentage was used for analysis. Plaque volumes (mm^3^) were calculated for each coronary lesion and then summated to compute the total plaque volume for the entire segment. Total per-patient coronary plaque volume was normalized to the total per–patient vessel volume to account for variation in coronary artery volume, calculated as: plaque volume/vessel volume × 100%, and reported as percentage atheroma volume (PAV). Plaque volume was categorized using HU ranges, with non-calcified plaque volume defined as HU between +30 and +350 and calcified plaque volume defined as >350 HU.^12^ The AI-QCT algorithm has been validated against expert CT readers, quantitative coronary angiography, and invasive fractional flow reserve.^4,5,13^ The AI-QCT tool has been shown to provide incremental prognostic information over clinical risk factors, the coronary calcium score, and the CAD-RADS reporting system.^14^ While the AI-QCT tool used has shown greater accuracy for DS% assessment compared with manual site readers or core labs, these findings come from different trials, limiting external validity.^15^ In addition, Cleerly-based plaque burden (PAV) quantification has been validated against manual CCTA-based plaque assessment, but less extensively against invasive reference standards such as IVUS or OCT.

### Follow-up

Follow-up was collected using electronic standardized telephonic follow-up, medical records, and national registry databases. The identified events were confirmed by investigators according to the guidelines of the European Society of Cardiology.^16^ For the current analysis, events included the occurrence of all-cause mortality or non-fatal myocardial infarction (MI), whichever occurred first. Deaths and periprocedural MIs related to early revascularization were not incorporated in the outcome analyses. If mortality and MI occurred on the same day, mortality was considered the patient’s event for the combined outcome. For analysis, the maximal follow-up time was 8 years.

### Statistical analysis

Continuous variables are expressed as mean ± SD or median (interquartile range) where appropriate. Categorical variables are presented as frequencies with percentage. The most severe DS% within a patient was used for analyses. Adjusted hazard ratios (aHRs) were determined using multiple Cox proportional hazard models incorporating the maximal DS%, PAV and cardiovascular risk factors at the time of the baseline CCTA [age, sex, body mass index (BMI), hypertension, hypercholesterolaemia, diabetes, and smoking status] with different follow-up times, i.e. at 0.5 year, 1 year and each following consecutive year until 8 years of follow-up. Patients with an event that occurred after the specific follow-up time were censored at that particular time. Differences between aHRs were statistically tested by comparison of 95% confidence intervals (CIs). Additionally, Cox proportional hazards regression models with a time-dependent covariate were used to assess whether DS% or PAV showed a different predictive value <1 year or from 1 to 8 years of follow-up. Time to event was calculated starting from CCTA imaging. Adjusted HRs are presented per unit increase, being per percentage increase for DS and PAV. Missing cardiovascular risk factors were multiple imputed using a fully conditional specification. A number of 20 imputed copies were created of the original data set and analysed, after which the results were pooled. Two sensitivity analyses were performed to correct for early revascularizations based upon the initial diagnostic workup. The first sensitivity analysis comprised the same Cox models as previously described but is adjusted for early revascularization status. The second sensitivity analysis excluded all patients with an early revascularization. A two-sided *P* value <0.05 was considered statistically significant. All statistical analyses were performed using IBM SPSS software package version 28 (IBM SPSS Statistics, IBM Corporation, Armonk, NY, USA).

## Results

**Figure 1:**
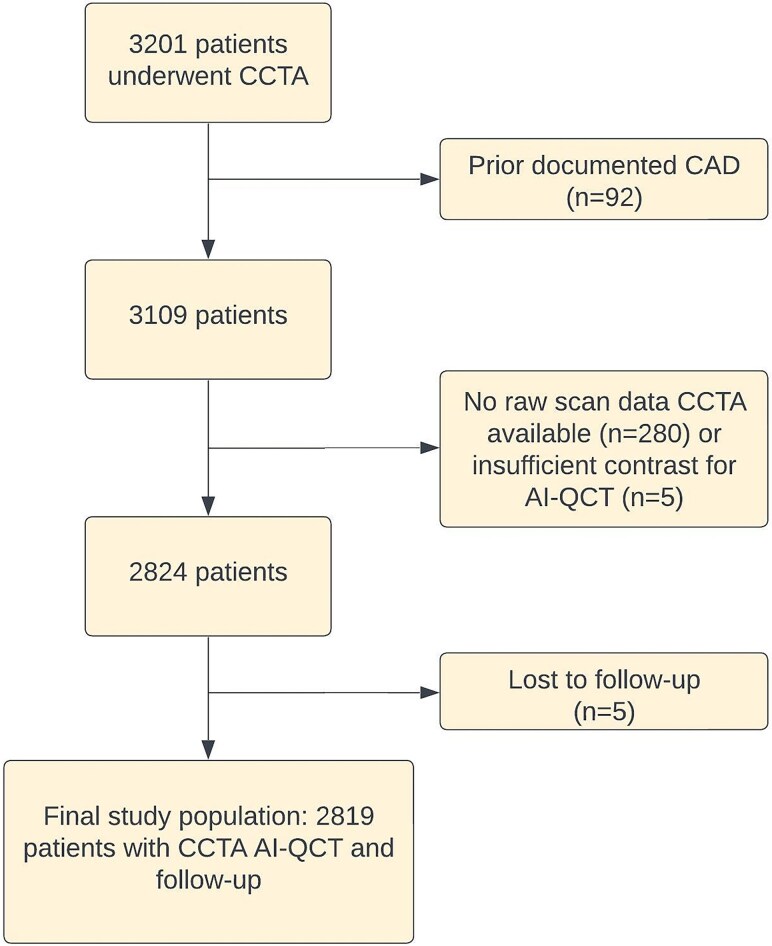
Patient selection flow chart. Abbreviations: AI-QCT, atherosclerosis imaging-quantitative coronary computed tomography; CCTA, coronary computed tomography angiography.

Of 3201 patients evaluated for inclusion, 92 were excluded because of prior documented CAD, 280 patients were excluded from analysis because the raw scan data were unavailable, in five patients, poor scan quality precluded AI-QCT analyses, while five patients were lost to follow-up (*Figure 1*). As such, the study population consisted of 2819 patients (*n* = 1563 female, 55%) with a mean age of 62 ± 10 years. The majority of patients was referred because of chest pain (80%). During a median follow-up of 6.9 (interquartile range 4.9–8.0) years, 235 events occurred. The events consisted of 160 deaths and 75 MIs. Baseline characteristics are depicted in *Table 1*. Patients who experienced an event exhibited significantly more severe stenoses and had a higher plaque burden compared to patients without an adverse event (*Table 2*). A total of 319 (11.3%) patients underwent an early revascularization prompted by the CCTA findings.

**Table 1:** Baseline characteristics

	All *n* = 2819
Male	1256 (45%)
Age, years	62 ± 10
BMI, kg/m^2^ (*n* = 2009)	28 ± 5
Diabetes mellitus (*n* = 2577)	444 (17%)
Hypertension (*n* = 2589)	1542 (60%)
Hypercholesterolemia (*n* = 2603)	1649 (63%)
Current smoker (*n* = 2617)	473 (18%)
Family history of CAD (*n* = 2302)	1366 (59%)
Antiplatelet therapy (*n* = 2521)	1413 (56%)
Beta-blocker (*n* = 2533)	1355 (54%)
Calcium channel blocker (*n* = 2501)	487 (17%)
ACE-inhibitor (*n* = 2508)	502 (20%)
ARB (*n* = 2512)	569 (23%)
Lipid lowering therapy (*n* = 2530)	1288 (51%)
Typical AP	693 (26%)
Atypical or aspecific AP	1487 (55%)
Other	538 (20%)

Abbreviations: AP, angina pectoris; BMI, body mass index; CAD, coronary artery disease; ACE, angiotensin-converting-enzyme; ARB, angiotensin II receptor blocker.

**Table 2:** CCTA findings

	All *n* = 2819	No event *n* = 2596	Event *n* = 223	*P*-value
**Stenosis grade**				
Diameter stenosis percentage	23 (9–51) %	21 (9–48) %	45 (25–65%) %	<0.01
≥50% diameter stenosis	752 (27%)	646 (25%)	106 (48%)	<0.01
**Plaque volumes**				
Percent atheroma volume	3.5 (1.1–10.0)	3.1 (1.0–9.1) %	12.0 (4.6–22.1) %	<0.01
Percent calcified plaque volume	0.6 (0.0–3.3)	0.4 (0.0–2.9) %	3.5 (0.9–9.2) %	<0.01
Percent non-calcified plaque volume	2.5 (0.9–6.3)	2.4 (0.9–5.8) %	6.7 (3.1–10.7) %	<0.01

CCTA findings, median with 95% confidence intervals.

### Short-term follow-up

**Figure 2:**
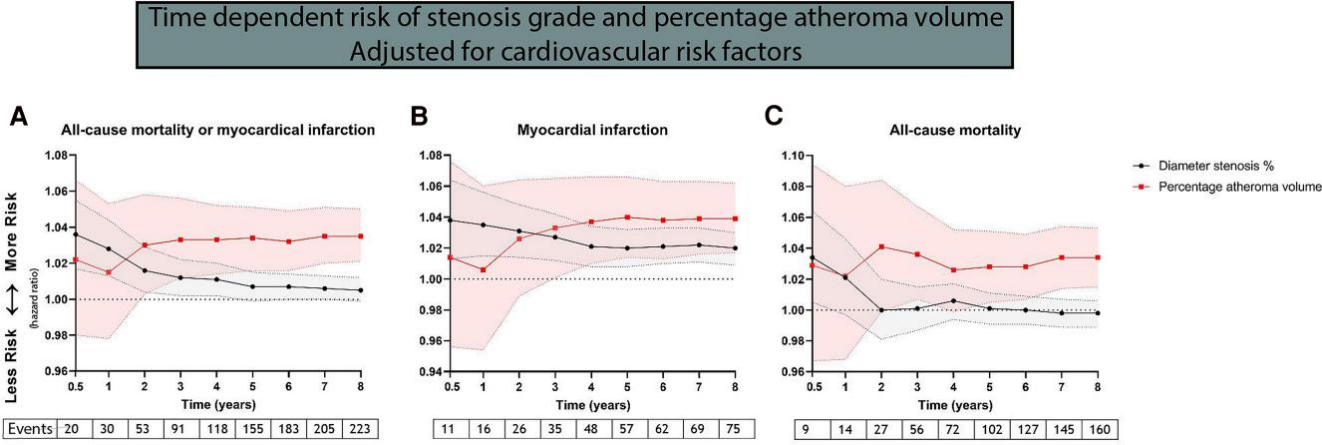
Time dependent risk of stenosis grade and percentage atheroma volume.

*Figure 2* illustrates the aHRs accounting for cardiovascular risk factors, DS% and PAV across a follow-up period ranging from 0.5 to 8 years. At 1-year follow-up, DS% was significantly predictive for the combined outcome (aHR 1.028, 95% CI 1.013–1.044) and the occurrence of MI (aHR 1.035, 95% CI 1.015–1.056), but not significantly predictive for mortality (aHR 1.021, 95% CI 0.997–1.046, *Figure 2*). The short-term event rate of patients with mild, moderate, and severe coronary stenoses is shown in Supplementary data online, *Figure S1*. In contrast, PAV did not significantly demonstrate predictive value for the combined outcome (1.015, 95% CI 0.978–1.053), MI (aHR 1.0060, 95% CI 0.9540–1.0600), or mortality (aHR 1.0340, 95% CI 1.0150–1.0530) at 1-year follow-up. Sensitivity analyses excluding or correcting for early revascularization indicated similar results (see Supplementary data online, *Table S1* and Supplementary data online, *Figure S2*).

### Long-term follow-up

At 8-year follow-up, DS% was not predictive for the combined outcome (mortality or MI, aHR 1.005, 95% CI 0.999–1.012) or mortality (aHR 0.998, 95% CI 0.989–1.006, *Figure 2*). However, DS% was a significant predictor for the occurrence of MI (aHR 1.020, 95% CI 1.009–1.030) at 8-year follow-up. Meanwhile, PAV was predictive for the combined outcome (aHR 1.035, 95% CI 1.021–1.050), mortality (1.034, 95% CI 1.0150–1.0530), and MI (1.039, 95% CI 1.017–1.0620) at 8-year follow-up. Sensitivity analyses excluding or correcting for early revascularization indicated similar results (see Supplementary data online, *Table S1* and Supplementary data online, *Figure S2*).

### Change of predictive value

**Figure 3:**
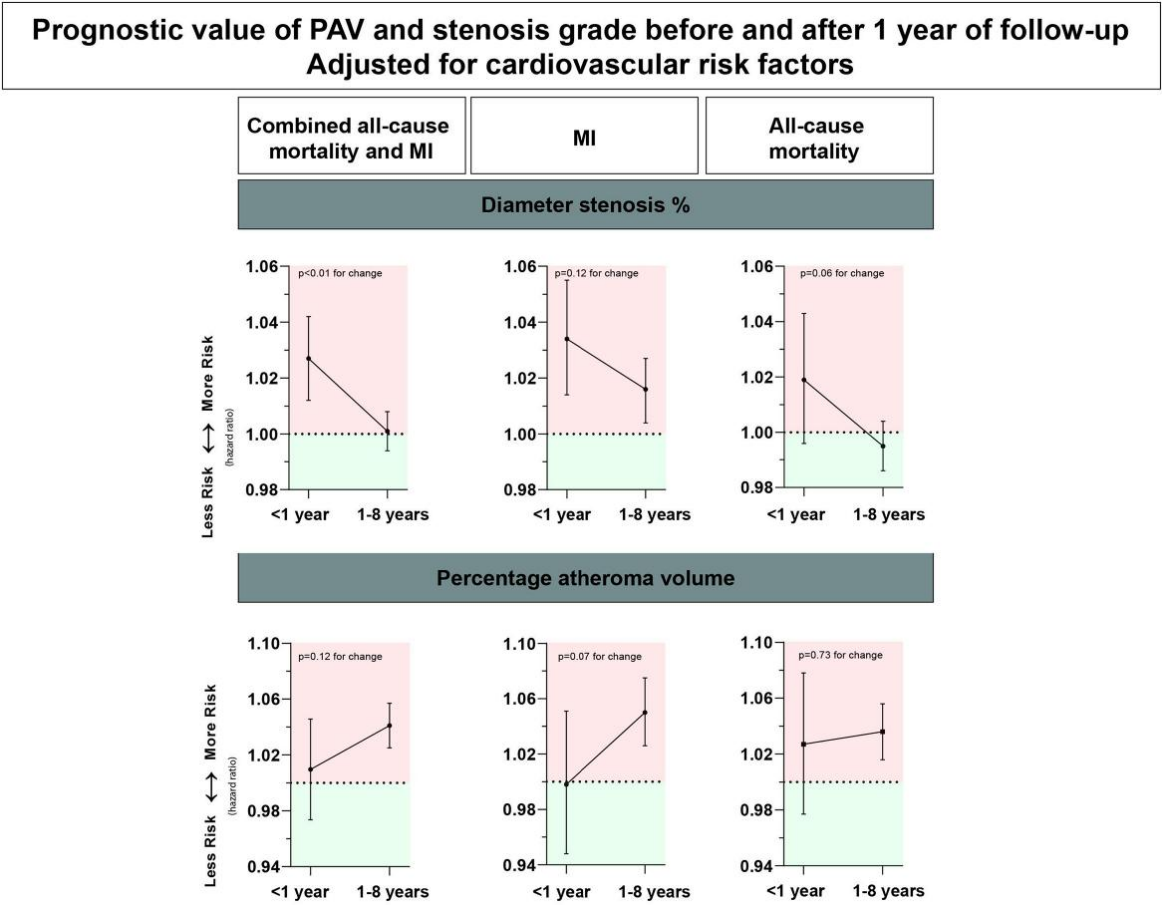
Change of prognostic value of stenosis grade and plaque burden before and after 1 year of follow-up. Abbreviations: MI, myocardial infarction; PAV, percentage atheroma volume.

The aHRs for PAV and DS% intersected between the first and second year of follow-up, suggesting a trend wherein PAV emerges as a more robust predictor of the combined outcome compared to DS% (*Figure 2*). By the fourth year of follow-up, PAV significantly outperformed DS% in predicting the combined outcome (aHR PAV: 1.036, 95% CI 1.007–1.067 vs. aHR DS%: 1.001, 95% CI 0.987–1.015) per per cent increase. Similar trends were observed for the prediction of MI and mortality (*Figure 2*). *Figure 3* illustrates aHRs before 1 year and from 1 to 8 years of follow-up were estimated with Cox proportional hazards regression models with a time-dependent covariate. The predictive value of DS% for combined mortality or MI was significantly stronger before 1 year than after 1 year of follow-up (aHR <1 years 1.027, 95% CI 1.012–1.042 vs. 1.001, 95% CI 0.994–1.008; *P* < 0.01 for difference in aHR). Stratification for MI and all-cause mortality did not reveal significant differences <1 year and between 1 and 8 years (MI, *P* = 0.12; mortality, *P* = 0.06 for difference). The predictive value of PAV for combined all-cause mortality or MI was not different before and after 1 year follow-up (aHR 1.009, 95% CI 0.974–1.046 and 1.041, 95% CI 1.025–1.057; *P* = 0.12). Stratification for MI and all-cause mortality did not reveal significant differences in terms of different predictive values < 1 year and between 1 and 8 years (MI, *P* = 0.07; mortality, *P* = 0.73 for difference). Sensitivity analyses including early revascularization status in the Cox model and sensitivity analyses excluding patients with early revascularization indicated similar results (see Supplementary data online, Table  *S1* and Supplementary data online, *F*igure  *S3*).

## Discussion

In this observational two-centre registry, the main findings are as follows: in terms of short-term follow-up (1 year), stenosis grade was a strong predictor of events, while plaque burden did not significantly demonstrate predictive value. In contrast, plaque burden was a robust predictor of long-term (8 years) events, whereas stenosis grade was not predictive for outcome on the long-term. Stenosis grade was a significantly stronger predictor on the short-term (<1 year) in comparison to the long-term (1–8 years), whereas no significant change was observed concerning the predictive power of plaque burden throughout follow-up.

Historically, obstructive CAD has been the primary focus in CAD management because of its central role in causing ischaemia.^17^ Therefore, initial studies evaluating the prognostic value of CCTA focused on DS.^2^ These studies demonstrated incrementally increasing adverse events for patients without CAD, with non-obstructive CAD (<50% DS), or with obstructive CAD (≥50% DS). However, in a nested case–control study comprising acute coronary syndrome and non-event patients, Chang *et al.* observed that, although stenosis grade was related to future acute coronary syndromes, three-fourths of culprit lesion precursors were non-obstructive (<50% DS) on baseline CCTA imaging.^3^ The authors highlighted that, next to stenosis severity, plaque burden emerged as an important predictor of future events. These findings are further substantiated by numerous CCTA studies, showing that events are not caused by obstructive CAD *per se*, but also related to the extent of CAD.^6–8,18^ In terms of predicting prognosis, conflicting results have been reported on the importance of stenosis grade and plaque burden. In a SCOT-HEART sub-study with a 5-year follow-up, stenosis grade (as a dichotomous variable) did not confer incremental prognostic value after adjustment for plaque burden.^7^ Yet, in a CONFIRM sub-study with a 2-year follow-up, stenosis grade contributed significantly to plaque burden for risk prediction. Our study provides rationale on how these apparent conflicting results may relate. In addition, in the main outcome paper of the SCOT-HEART trial, the reduced event rate in the CCTA arm was primarily driven by a decrease in spontaneous MI at 5-year follow-up.^19^ In our study, both PAV and DS% were predictive of MI at 5 years, whereas their association with all-cause mortality was borderline or not significant. In this respect, our findings are consistent with those of SCOT-HEART. In our study, the relationship between PAV and all-cause mortality becomes stronger beyond 5 years. Thus, if preventive therapies continue to exert long-term effects on plaque composition and progression, a CCTA-first strategy may ultimately translate into mortality reduction, though likely only after longer follow-up.

Our findings reflect the situation at the time of baseline CCTA. Although it is challenging to capture plaque immediately prior to MI, a certain dynamicity in plaque phenotype and DS% has been observed, with a rapid accumulation and increasing stenosis grade before MI.^20,21^ Importantly, contemporary results of registries might underestimate the true effect of baseline stenosis grade, as severe stenoses will most probably have been frequently revascularized. Notably, in the present study, our findings remained consistent after exclusion of patients with early revascularizations. It deserves consideration that Cox proportional hazard models utilize the cumulative sum of events for the calculation of aHR. This signifies (in accordance with prior literature) that in models with extended follow-up periods, all events occurring from baseline CCTA until the end of the follow-up period (maximum 8 years) are included, rather than only late events.

Because manual plaque quantification is a time-intensive process, multiple machine-learning software tools have recently been developed to rapidly and reproducibly quantify coronary plaque burden.^4,5,7,22^ These software tools facilitate studies with a correction for coronary plaque itself rather than derivatives, although useful, such as the segment involvement score.^23^ As such, our study assesses the predictive value of stenosis grade and plaque burden per percentage increase, rather than utilizing arbitrary cut-offs for either index with a concomitant loss of information. The prognostic capacity of AI-QCT has been further demonstrated, with studies showing predictive value beyond the coronary artery calcium score.^24^ However, to become a meaningful tool in CAD management, AI-QCT must not only predict coronary events but also influence clinical decision-making. In this context, AI-QCT–derived information may encourage the initiation of preventive therapies over standard CCTA reports, yet it remains uncertain to what extent such therapies are actually prescribed following AI-QCT assessment.^25^

Our findings might facilitate physicians as well as software application tools to more accurately identify patients at risk for cardiovascular events. Nonetheless, the current results should be interpreted in the context of a systemic lipid-driven inflammatory disease with focal spots of increased plaque accumulation, potentially resulting in high-grade stenosis.^26^ Our findings imply that use of a single index of CAD might not effectively capture an individual’s risk for future events at a single time point. Moreover, future machine learning risk prediction software tools should utilize the dynamic risk profiles associated with stenosis grade and plaque burden to predict risk at different points in time. A truly comprehensive risk assessment would combine time-dependent clinical factors, such as age and BMI, with imaging findings to more accurately reflect a patient’s evolving risk profile.

## Study limitations

Several limitations of this study deserve consideration. First, for AI-QCT quality assurance adjustment, several segments had to be excluded because of poor image quality, which may have affected the present findings. Secondly, a limited number of events occurred at short-term follow-up, which might affect the aHRs with accompanying 95% CI. Thirdly, cause of death was not known, and therefore all-cause mortality was used for analysis. Although all-cause mortality is not a direct cardiac endpoint, it is in contrast to cardiac mortality not affected by verification bias.^27^ In addition, we observed comparable time-dependent trends for all-cause mortality and MI, which lend support to our findings. Fourthly, type and intensity of medical treatment following CCTA were not available, while the SCOT-HEART demonstrated the prognostic relevance of medication intensification following cardiac imaging.^19^ Finally, the relatively small number of short-term events in our study precluded meaningful subgroup analyses.

## Conclusion

Stenosis grade holds the highest prognostic significance for short-term cardiovascular events, while a significant trend was observed showing a diminishing predictive capacity for long-term events. Plaque burden demonstrated robust long-term predictive value.

## Supplementary Material

jeag022_Supplementary_Data

## Data Availability

Data available on request.
