# Supplementary material for: The time-varying prognostic value of stenosis and plaque burden in coronary artery disease
Source: Eur Heart J Cardiovasc Imaging. 2026 Jan 28;27(4):696–703. doi: 10.1093/ehjci/jeag022 (PMC13021277; doi:10.1093/ehjci/jeag022)

**Supplemental table 1.** Adjusted hazard ratios corrected for risk factors and early revascularization

| Follow-up time (years) | DS% (95% CI) | p-value | PAV  (95% CI) | p-value | Early revascularization  (95% CI) | p-value |  |
| --- | --- | --- | --- | --- | --- | --- | --- |
| All-cause mortality or myocardial infarction | | | | | | |  |
| 0.5 | 1.040 (1.020-1.061) | <0.01 | 1.024 (0.981-1.069) | 0.28 | 0.568 (0.189-1.701) | 0.31 |  |
| 1 | 1.027 (1.011-1.044) | <0.01 | 1.014 (0.977-1.053) | 0.46 | 1.128 (0.453-2.808) | 0.80 |  |
| 2 | 1.017 (1.003-1.030) | 0.01 | 1.017 (1.003-1.030) | 0.03 | 0.967 (0.478-1.956) | 0.93 |  |
| 3 | 1.015 (1.004-1.025) | <0.01 | 1.036 (1.014-1.058) | <0.01 | 0.576 (0.317-1.048) | 0.07 |  |
| 4 | 1.013 (1.004-1.022) | <0.01 | 1.035 (1.015-1.055) | <0.01 | 0.687 (0.407-1.162) | 0.16 |  |
| 5 | 1.009 (1.000-1.017) | 0.04 | 1.035 (1.018-1.053) | <0.01 | 0.776 (0.487-1.237) | 0.29 |  |
| 6 | 1.008 (1.001-1.016) | 0.04 | 1.033 (1.017-1.050) | <0.01 | 0.854 (0.557-1.309) | 0.47 |  |
| 7 | 1.008 (1.000-1.015) | 0.04 | 1.037 (1.021–1.052) | <0.01 | 0.805 (0.536-1.208) | 0.30 |  |
| 8 | 1.006 (1.000-1.013) | 0.07 | 1.037 (1.022-1.052) | <0.01 | 0.787 (0.529-1.172) | 0.24 |  |
| Myocardial infarction | | | | | | | |
| 0.5 | 1.041 (1.013-1.069) | <0.01 | 1.016 (0.957-1.078) | 0.60 | 0.747 (0.175-3.193) | 0.69 |  |
| 1 | 1.027 (1.003-1.050) | 0.03 | 1.027 (1.003-1.050) | 0.94 | 2.567 (0.704-9.356) | 0.15 |  |
| 2 | 1.028 (1.010-1.047) | <0.01 | 1.024 (0.987-1.062) | 0.20 | 1.385 (0.543-3.528) | 0.50 |  |
| 3 | 1.028 (1.012-1.044) | <0.01 | 1.034 (1.002-1.066) | 0.04 | 0.792 (0.344-1.826) | 0.59 |  |
| 4 | 1.021 (1.007-1.035) | <0.01 | 1.038 (1.010-1.065) | <0.01 | 0.916 (0.434-1.933) | 0.82 |  |
| 5 | 1.020 (1.007-1.033) | <0.01 | 1.040 (1.014-1.065) | <0.01 | 0.998 (0.506-1.971) | 0.97 |  |
| 6 | 1.021 (1.009-1.034) | <0.01 | 1.038 (1.013-1.063) | <0.01 | 1.011 (0.532-1.921) | 0.97 |  |
| 7 | 1.022 (1.011-1.034) | <0.01 | 1.040 (1.016-1.063) | <0.01 | 0.974 (0.532-1.785) | 0.93 |  |
| 8 | 1.020 (1.009-1.031) | <0.01 | 1.039 (1.017-1.062) | <0.01 | 1.013 (0.562-1.825) | 0.97 |  |
| All-cause mortality | | | | | | | |
| 0.5 | 1.040 (1.010-1.071) | 0.01 | 1.031 (0.968-1.098) | 0.34 | 0.406 (0.072-2.298) | 0.31 |  |
| 1 | 1.026 (1.001-1.052) | 0.04 | 1.026 (0.970-1.085) | 0.37 | 0.377 (0.074-1.918) | 0.24 |  |
| 2 | 1.003 (0.983-1.024) | 0.76 | 1.043 (1.001-1.087) | 0.04 | 0.549 (0.168-1.795) | 0.32 |  |
| 3 | 1.005 (0.991-1.019) | 0.52 | 1.041 (1.011-1.072) | <0.01 | 0.375 (0.149-0.942) | 0.04 |  |
| 4 | 1.009 (0.997-1.021) | 0.15 | 1.030 (1.003-1.057) | 0.03 | 0.442 (0.205-0.955) | 0.04 |  |
| 5 | 1.003 (0.992-1.013) | 0.62 | 1.030 (1.007-1.054) | 0.01 | 0.655 (0.351-1.222) | 0.18 |  |
| 6 | 1.001 (0.992-1.011) | 0.79 | 1.030 (1.008-1.051) | <0.01 | 0.734 (0.420-1.283) | 0.28 |  |
| 7 | 1.000 (0.991-1.009) | 0.92 | 1.036 (1.016-1.056) | <0.01 | 0.692 (0.408-1.174) | 0.17 |  |
| 8 | 1.000 (0.991-1.008) | 0.94 | 1.037 (1.018-1.056) | <0.01 | 0.621 (0.370-1.044) | 0.07 |  |

**Included in the model:** cardiovascular risk factors, DS%, PAV and early revascularization status

**Abbreviations:** CI, confidence interval; DS%, diameter stenosis; PAV, percentage atheroma volume

**Supplemental figure 1.** Short-term event rate of patients with mild, moderate or severe coronary stenosis.


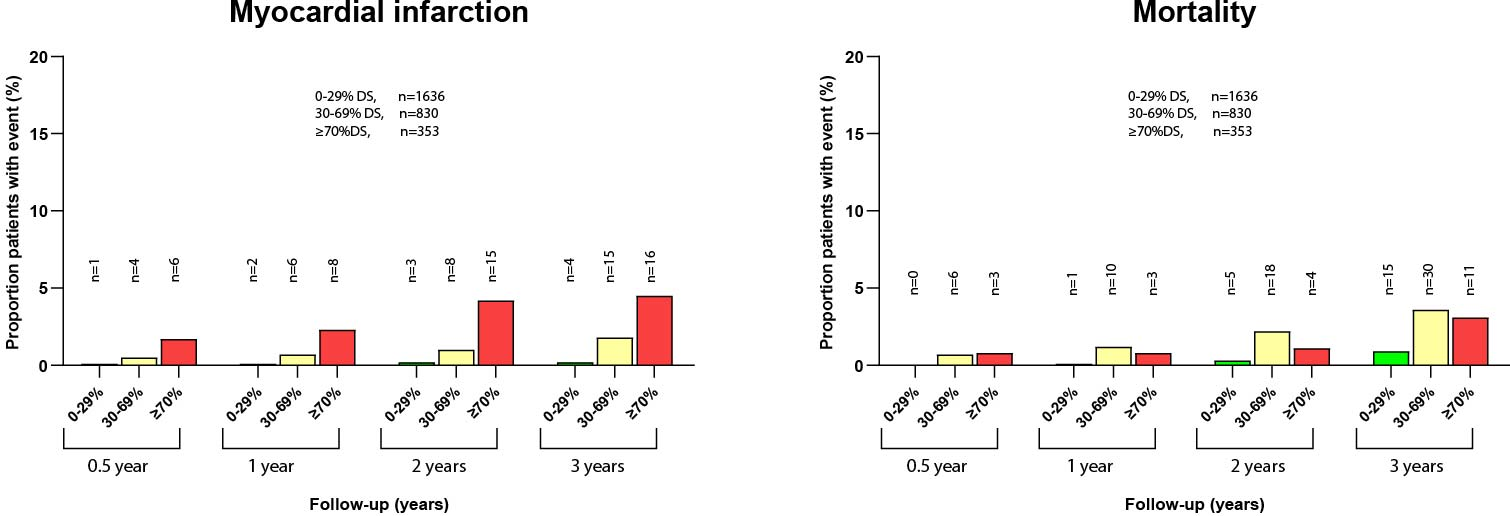


**Supplemental figure 2.** The time dependent risk of stenosis grade and plaque burden in patients without early revascularization


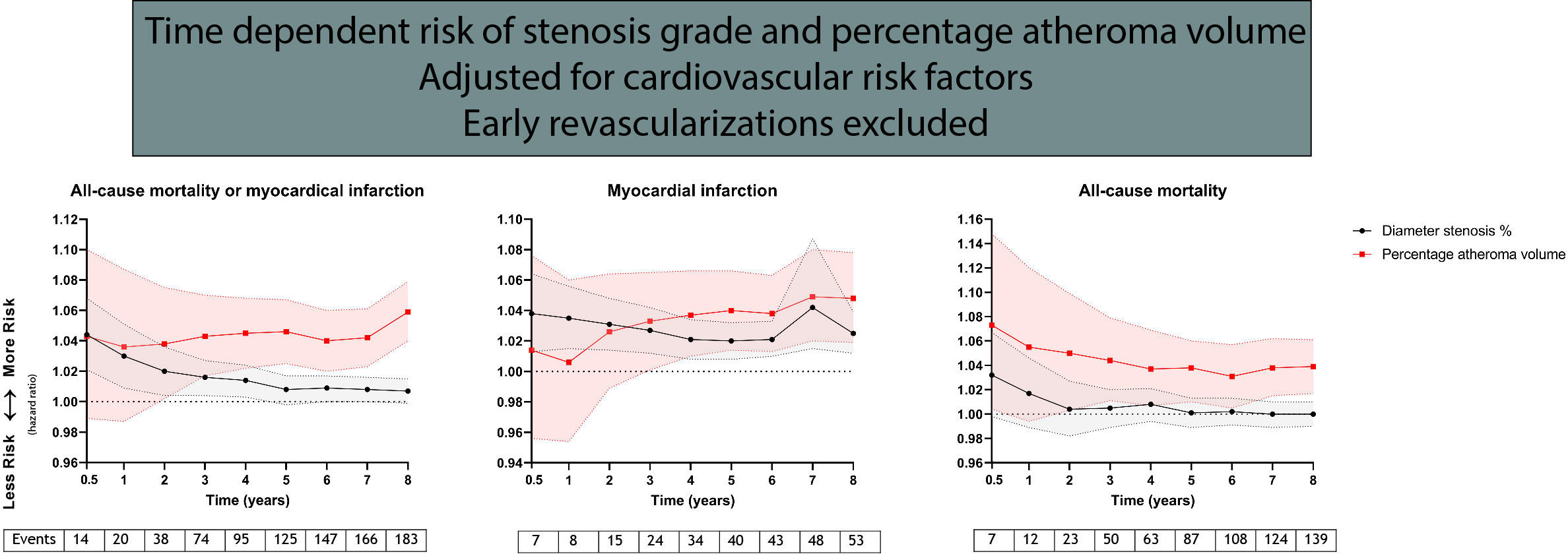


**Supplemental figure 3.** The prognostic value of stenosis grade and plaque burden before and after 1 year of follow-up in patients without early revascularization


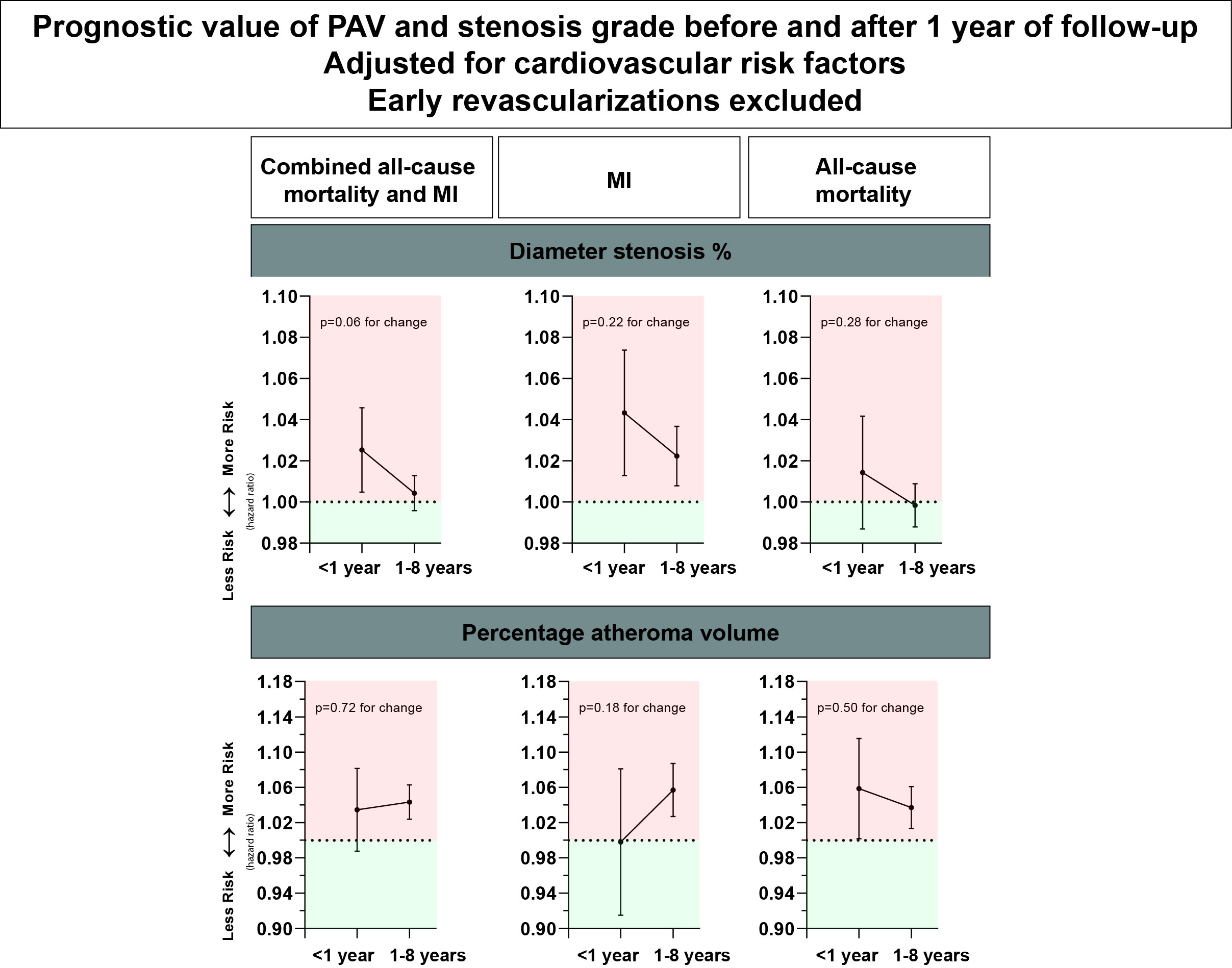

Supplement: jeag022_Supplementary_Data [file jeag022_supplementary_data.docx]
